# Dietary diosgenin transcriptionally down-regulated intestinal NPC1L1 expression to prevent cholesterol gallstone formation in mice

**DOI:** 10.1186/s12929-023-00933-3

**Published:** 2023-06-27

**Authors:** Weiyi Shen, Wentao Shao, Qihan Wang, Bo Wang, Gang Zhao, Aihua Gu, Zhaoyan Jiang, Hai Hu

**Affiliations:** 1 Center of Gallstone Disease, Shanghai East Hospital, and Institution of Gallstone Disease, School of Medicine, Tongji University, Shanghai, China; 2 State Key Laboratory of Reproductive Medicine, School of Public Health, Nanjing Medical University, Nanjing, Jiangsu China; 3 Collaborative Innovation Center for Cardiovascular Disease Translational Medicine, Center for Global Health, Nanjing Medical University, Nanjing, Jiangsu China

**Keywords:** Gallstone, Diosgenin, Cholesterol, NPC1L1, Microbiome

## Abstract

**Background:**

Cholesterol gallstone disease is a common disease. Reducing cholesterol burden is important to prevent/treat gallstone. In this study, we investigated the application of diosgenin (DG) to prevent the formation of gallstone in mice.

**Methods:**

Adult male C57BL/6J mice were fed with the lithogenic diet (LD) only or LD supplemented with DG or ezetimibe for 8 weeks. Incidences of gallstone formation were documented. Intestine and liver tissues were collected to measure the lipid contents and expression of genes in cholesterol metabolism. Caco2 cells were treated with DG to monitor the regulation on cholesterol absorption and the transcriptional regulation of *Npc1l1* gene. Changes of gut microbiota by DG was analyzed. Intraperitoneal injection of LPS on mice was performed to verify its effects on STAT3 activation and *Npc1l1* expression in the small intestine.

**Results:**

LD led to 100% formation of gallstones in mice. In comparison, dietary DG or ezetimibe supplementary completely prevents gallstones formation. DG inhibited intestinal cholesterol absorption in mice as well as in Caco2 cells by down-regulation of *Npc1l1* expression. DG could directly inhibit phosphorylation of STAT3 and its transcriptional regulation of *Npc1l1* expression. Furthermore, DG could modulate gut microbiota profiles and LPS mediated STAT3 activation and *Npc1l1* expression.

**Conclusion:**

Our results demonstrated that dietary DG could inhibit intestinal cholesterol absorption through decreasing NPC1L1 expression to prevent cholesterol gallstone formation.

**Supplementary Information:**

The online version contains supplementary material available at 10.1186/s12929-023-00933-3.

## Highlights

1. Diosgenin prevented cholesterol gallstone formation.
2. Diosgenin could directed inhibited of STAT3 mediated transcription of *Npc1l1* gene.
3. Diosgenin could also modulate gut microbiota-LPS mediated STAT3 activation.
4. Diosgenin inhibited intestinal cholesterol absorption, thereby, lowered cholesterol in liver and bile.

**Supplementary Information:**

The online version contains supplementary material available at 10.1186/s12929-023-00933-3.

## Introduction

Gallstone disease is a prevalent disease world-widely. About 20% of the European population [1], 16.5% in the United States [2] and > 10% in China [3] suffer from gallstone. Majority (> 90%) of gallstones are composed of cholesterol [4]. Supersaturation of biliary cholesterol is known to be a prerequisite for gallstone formation [5]. Gut-derived excess dietary cholesterol is an important source of biliary cholesterol [6]. Therefore, inhibition of intestinal cholesterol absorption is considered to be a promising therapeutic measure to prevent gallstone formation. So far, ezetimibe is one prescribed drug to inhibit intestinal cholesterol absorption by targeting Niemann-Pick C1-like 1 (NPC1L1), an identified protein for cholesterol transportation at the intestinal epithelium [7]. Ezetimibe has been proved to be effective in preventing cholesterol gallstone formation in mice [8]. Certain herbal medicine and their extracts have been proposed in gallstone prevention as well [8, 9]. For example, phytosterols, analogs of cholesterol, extracted from grains, nuts and oil, exhibit ability to reduce cholesterol absorption and further prevent gallstones [10].

Diosgenin (DG, C_27_H_42_O_3_) is a natural steroidal sapogenin, which is widely found in wild yam and other dioscorea plants. The DG content in several yam species varies considerably, which ranges from 0.78 mg/g to 19.52 mg/g dry weight [11]. The main structure of DG is composed of steroid ring and lacks hydrophilic groups, which leads to its hydrophobicity and low bioavailability [12]. Although DG is usually applied as production precursor for most hormonal drugs, DG itself has wide range of promising biological applications [12]. Complementary therapy of cardiovascular disease and tumor are the two most proposed fields of DG use [13, 14].

Some previous studies showed the anti-atherosclerosis effect of DG partly by reducing serum cholesterol level [13, 15]. The specific pathway that DG lowers cholesterol is currently inconclusive. Early studies suggested that DG competed with cholesterol to form micelles, resulting in less cholesterol to be absorbed in the gut [15]. Accumulated evidence reveals that DG has multiple regulatory pathways to lower serum cholesterol level. DG was shown to ameliorate liver lipids metabolism via SREBP repression [16] which is an upstream regulator of cholesterol metabolism. Others found that DG inhibits intestine NPC1L1 and enhancing ABCG5 and ABCG8 directly, thereby enhancing cholesterol excretion by intestinal absorptive epithelium [17]. The cholesterol-lowering effect of DG may also be related to the elevation of ABCA1 and SRB1 [15]. These findings collectively suggested DG to be a promising dietary supplement targeting intestine to lower cholesterol. In this study, we aimed to investigate its effect on gallstone formation in mice and to identify the underlying mechanism as well.

## Materials and methods

### Animals and diets

SPF eight-week-old male C57BL/6J mice were purchased from Nanjing GemPharmatech Co. Ltd (Nanjing, China). The diosgenin powder (D101265, 95% pure) used in diet was purchased from Aladdin (Shanghai, China). All study procedures were approved by the Animal Ethics Committee of Shanghai East Hospital, School of Medicine, Tongji University, and performed in strict compliance with the Guidelines for Care and Use of Laboratory Animals  at School of Medicine, Tongji University. A total of 60 mice were randomly divided into 4 groups after 1 week of acclimation to the environment. LD group were fed with the lithogenic diet which contained 0.5% cholesterol and 0.25% cholic acid. Mice in LD-1DG group were fed with lithogenic diet and 1% diosgenin, that in LD-2DG group with lithogenic diet and 2% diosgenin, and that in LD-EZM with lithogenic diet and ezetimibe (5 mg/kg weight/day). Water and food were available ad libitum. The body weight of the mice was recorded weekly. After 8 weeks feeding and 12 h fasting, mice were anesthetized with avertin. Serum, liver, gallbladder, intestines and feces samples were collected, immediately frozen in liquid nitrogen and stored at − 80 ℃ until analysis. The quantification of gallstone was performed according to the method provided by Akiyoshi et al. (PMID: 3783046) when the gallbladder was removed.

Twenty mice were fed with 4 dietary models mentioned above (5 mice/group) for 2 weeks for measuring hepatic bile secretion. Following anesthesia, fresh bile secreted from the liver was collected within 60 min using UT-03 tubes (Unique Medical Co. Ltd, Japan).

Ten mice received corn oil + diosgenin (20 mg/mL) or corn oil alone daily by gavage, and proximal small intestine tissues were collected after 1 week. Another 10 mice were used to study the effects of LPS on the intestine, 5 mice were intraperitoneally injected with LPS (L2630, Sigma, Germany) of 10 mg/kg body weight, and the control group was injected with PBS. Serum and proximal small intestine were collected after 8 h.

### Lipid analysis

Bile, homogenized liver and feces were used to extracted lipids using FOLCH (chloroform:methonal-2:1) as described previously [18] and enzymatically measured using commercially available kits (Cholesterol: E1015, Applygen, China; Triglyceride: E1013, Applygen, China; Bile acids: BI7710, Zhicheng Biological Technology, China; Phospholipid: EFR0178, FUJIFILM, Japan). All procedures were carried out in accordance to the instructions by kits.

### Cell culture

Caco-2 cells were maintained in 25cm^2^ cell culture flask with Minimum Essential Medium (MEM) supplemented with 20% fetal bovine serum, 1% glutamax, 1 mM sodium pyruvate and Non-essential Amino Acids (11140050, Invitrogen, USA) and cultured at 37 °C and 5% CO2. Cells were preco-cultured with 5/10 μM DG with/without 25 μM cholesterol for 24 h. Diosgenin for cell culture was purchased from MCE (HY-N0177).

### Total RNA extraction and gene expression determination

Total RNA was isolated from the first third of the small intestines, the liver and Caco-2 cells using TRIzol Reagent (Invitrogen, Carlsbad, CA, USA). The total RNA concentration was adjusted to 1 μg in a 20 μl system. After single-stranded cDNA synthesized, the gene expression was quantified by quantitative real-time PCR (qRT-PCR) using SYBR Green Master Mix (4,344,463, Thermo Fisher Scientific, USA) on ABI QuantStudio6 Q6. The primer sequences are shown in the Table 1. Gapdh was used as the reference gene, and the amount of target mRNA was calculated by the ΔΔCt method.

**Table 1:** The primer sequence involved in the experiment

Gapdh-F	TGTGTCCGTCGTGGATCTGA
Gapdh-R	CCTGCTTCACCACCTTCTTGAT
Abcg5-F	AATGCTGTGAATCTGTTTCCCA
Abcg5-R	CCACTTATGATACAGGCCATCCT
Cyp7a1-F	AGCAACTAAACAACCTGCCAGTACTA
Cyp7a1-R	GTCCGGATATTCAAGGATGCA
Cyp2c70-F	TGGCTTTCTCAGCAGGAAGAA
Cyp2c70-R	AACTGGCTTGGTGTCGATGT
Hmgcr-F	CTTGTGGAATGCCTTGTGATTG
Hmgcr-R	AGCCGAAGCAGCACATGAT
Abcg8-F	TGCCCACCTTCCACATGTC
Abcg8-R	ATGAAGCCGGCAGTAAGGTAGA
Npc1l1-F	ATC CTC ATC CTG GGC TTT GC
Npc1l1-R	GCA AGG TGA TCA GGA GGT TGA
Srb1-F	CGG GAG CGT GGA CCC TAT GT
Srb1-R	ACA CGG TGT CGT TGT CAT TGA
Ldlr-F	GCATCAGCTTGGACAAGGTGT
Ldlr-R	GGGAACAGCCACCATTGTTG
Cyp8-F	GAACTCAACCAGGCCATGCT
Cyp8-R	GGCACCCAGACTCGAACCT
Cyp27a1-F	GCCTTGCACAAGGAAGTGACT
Cyp27a1-R	CGCAGGGTCTCCTTAATCACA
Abcb4-F	CGGCGACTTTGAACTAGGCA
Abcb4-R	CAGAGTATCGGAACAGTGTCAAC
Abca1-F	CCTGCTAAAATACCGGCAAGG
Abca1-R	AGTAACCCGTTCCCAACTGGT
HuNPC1L1-F	CTTCAGATGGCCAGGTTTTAGC
HuNPC1L1-R	TGTAATCCTGTGAGTTTTTCAGGG
HuHMGCR-F	ATAGGAGGCTACAACGCCCAT
HuHMGCR-R	TTCTGTGCTGCATCCTGTCC
HuABCA1-F	CCTGTTTCCGTTACCCGACTC
HuABCA1-R	ACAGGCGAGCCACAATGG
HuGAPDH-F	TGACAACTTTGGTATCGTGGAAGG
HuGAPDH-R	AGGCAGGGATGATGTTCTGGAGAG
CHIP1-F	GCCCCCATTACCAAAGCTGA
CHIP1-R	GTAGGTGGCCACGCAGG
C-FOS-F	ACTGCACCCTCGGTGTTGG
C-FOS-R	TGCTGACGCAGATGTCCTAAT
NPC1L1-1	TGTCCCCGCCTATACAATGG
	CCTTGGTGATAGACAGGCTACTG
NPC1L1-2	CTCTGCCCTCTGCAATGCTC
	GAACAGGCTGCCGAGTCTT
NPC1L1-3	CGCCCTTCTTTCTACATGGGT
	GAATCTGCGCTTACGAGGGAG
NPC1L1-4	ATCCTCATCCTGGGCTTTGC
	GCAAGGTGATCAGGAGGTTGA
c-fos	CGGGTTTCAACGCCGACTA
	TTGGCACTAGAGACGGACAGA

### Western blot

The frozen proximal small intestine and Caco-2 cells was homogenized in RIPA (P0013B, Beyotime Biotechnology, China) and 1 mM PMSF. The protein was extracted according to the reagent instructions. Prepared proteins were separated in a 10% SDS-PAGE gel and then transferred to PVDF membrane. The subsequent experimental protocol followed the instructions for primary (PA1-16800, Invitrogen; 9145S, CST; 30835S, CST) and secondary antibodies (YFSA02, YIFEIXUE Biotechnology, China).

### NBD-cholesterol uptake

NBD-cholesterol were purchased from SIGMA (A2006133) and diluted to 2 μg/ml as working solution. Adherent Caco2 cells were cultured in 10 μM diosgenin and NBD-cholesterol for 4 h and rinsed with adequate PBS three times. Hoechst 33342 (B2261, Sigma, Germany) was used for nuclear staining. For ex vivo model, 9 mice were fed with LD, LD-2DG or LD with ezetimibe diet; and intestinal villi were collected and assayed following the established method [19]. In brief, the duodenum was rinsed in cold PBS and digested with 30 mM EDTA; then the villi were sorted using a 70uM filter. The fluorescence images were processed by ImageJ software to calculate the mean fluorescence intensity (MFI). Images after splitting channels were selected the cell or villus region according to the threshold algorithm, and calculates the MFI based on the gray value and area.

### Molecular docking

The docking procedure was performed with AutoDock 4.2.6 program. Lamarckian Genetic Algorithm was applied for doing the conformation search. The 3D structure of STAT3 protein was gathered from RCSB Protein Data Bank (ID: 6tlc), and the structures of DG from PubChem database (ID: 99474). Results are presented in terms of binding energy (kcal/mol) and visualized by the PyMOL program.

### Dual-luciferase reporter assay

The NPC1L1 promoter sequences with the highest binding score with STAT3 on JASPAR were cloned into the pmirGLO luciferase reporter plasmid. The vector was transfected into 293T cells with Lipo3000 system. 24 h after transfection, diosgenin was added into medium and cultured with another 24 h until the cell was harvested. The Duo-Lite Luciferase Assay Kit (DD1205-01, Vazyme, China) was used to detect relative luciferase activity.

### Chromatin immune-precipitation assay (ChIP)

The ChIP assay for Caco2 cells was performed with SimpleChIP Assay Kit (#9005, Cell Signaling Technology) and Stat3 Rabbit mAb (#12640, Cell Signaling Technology). Micrococcal nuclease was applied to chromatin digestion; for getting appropriate size chromatin fragments, ultrasonic homogenizer was used with 30% intensity, 15 sets of 4 s pulses. The qPCR primers were designed according NC_000007.14 (44542155–44542250). The CHIP-Seq data of STAT3 was from published data [20].

### RNA-seq and gut microbiota analysis

The cecal contents were collected for the 16S rRNA sequencing. Sequencing and data analysis protocol were described previously [21]. In brief, sequencing data was obtained from Illumina miseq platform according to standard protocols. Raw data were clustered into OTUs (operational taxonomic unit) according to 97% similarity and then compared with the database for species annotation of OTUs. The raw data can be found on SRA (ID: PRJNA922754 and PRJNA923121).

### Statistics and figure

All data are expressed as the mean ± standard error of the mean (SEM). Significant differences were determined for the values among 4 groups by ANOVA and LSD-t test as post-hoc analysis. P < 0.05 indicated a statistically significant difference. Statistics were performed using SPSS 23.0. Differential gene enrichment for transcription factors was done through the online website (http://www.licpathway.net/KnockTF/), genes with FDR < 0.01 were input. Figures in this article are drawn by Figdraw and GraphPad Prism 8.

## Results

### Diosgenin (DG) prevented gallstone occurrence and ameliorate hepatic lipids deposition

**Fig. 1:**
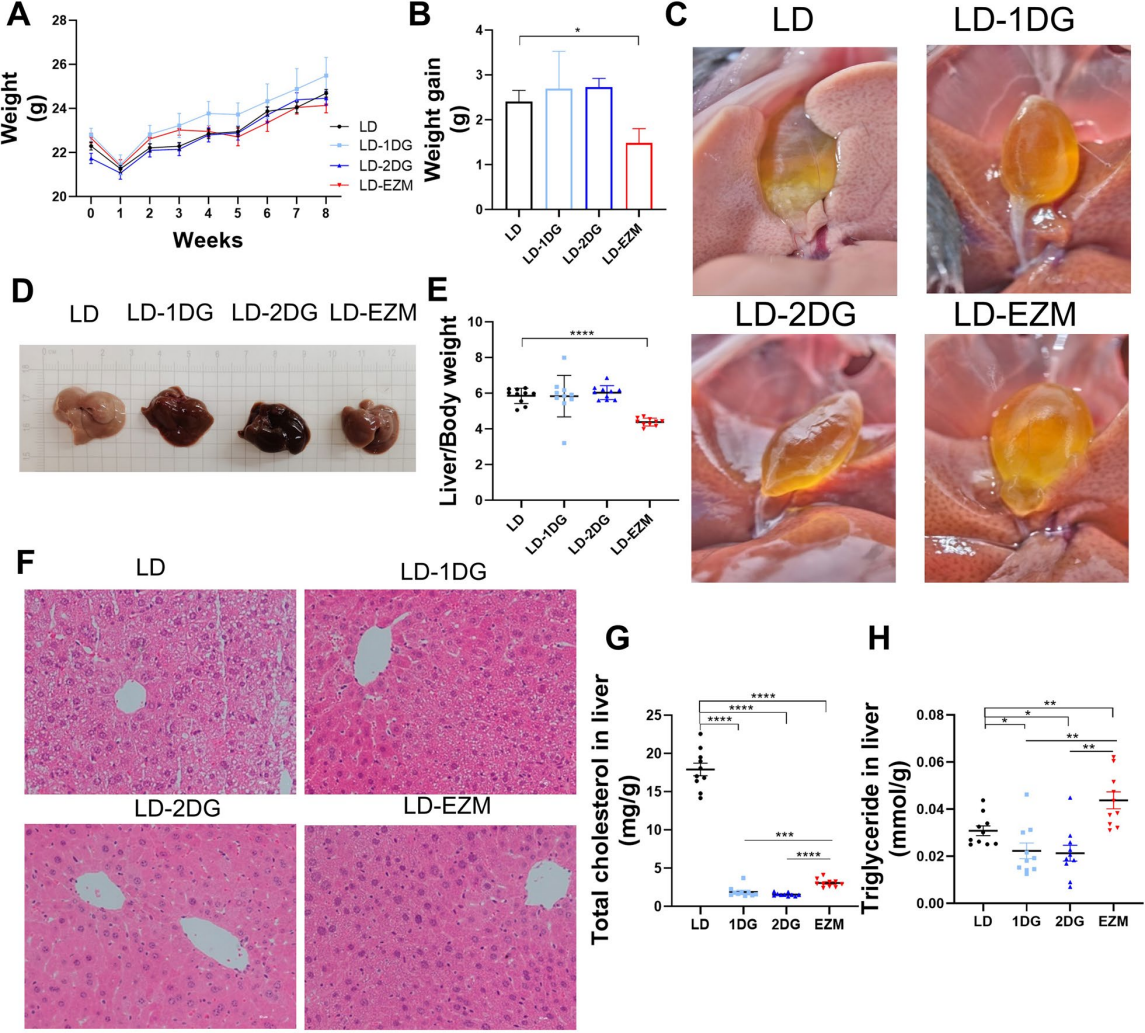
Diosgenin (DG) prevented cholesterol gallstone formation and alleviated hepatic lipid deposition. **A** Changes in the body weights of the mice fed with lithogenic diet (LD) during 8 weeks. **B** Body weight gain at 8 weeks. **C** Appearance of gallbladder in mice from LD, LD-1DG, LD-2DG and LD-EZM groups. Gallstone formed in 100% of mice fed with LD, but none in mice fed with LD-1DG, LD-2DG or LD-EZM. **D** Appearance of liver. **E** Ratio of liver to body weight. **F** Representative H&E staining of liver tissues from the four groups. **G** and **H** Hepatic total cholesterol and triglyceride levels, respectively. Data are expressed as mean ± SEM (n = 10 in each group). *P < 0.05, **P < 0.01, ***P < 0.001, ****P < 0.0001. LD: lithogenic diet; LD-1DG: LD + 1% diosgenin; LD-2DG: LD + 2% diosgenin; LD-EZM: LD + ezetimibe

DG or ezetimibe in diet led to a slight but insignificant decrease in body weight in mice (Fig. 1A and B). Gallstone formation in 100% of the mice fed with LD only. In contrast, none gallstone (0%) formed in mice fed with LD supplemented with either dose of DG (LD-1DG and LD-2DG) or ezetimibe (LD-EZM) (Fig. 1C).

Livers from mice in LD-1DG and LD-2DG groups appeared to be ruddier than that from mice in LD groups (Fig. 1D). The liver index did not differ between mice in LD-DG and LD groups, but was lower in LD-EZM group (Fig. 1E). HE staining showed more vacuoles in hepatocytes in the LD group than that in the rest groups. DG or ezetimibe obviously alleviated pathological changes in liver tissues (Fig. 1F). Hepatic cholesterol levels significantly decreased in both DG and ezetimibe groups (Fig. 1G). Hepatic triglycerides were reduced only in the DG groups (Fig. 1H).

### Diosgenin decreased biliary cholesterol saturation

**Fig. 2:**
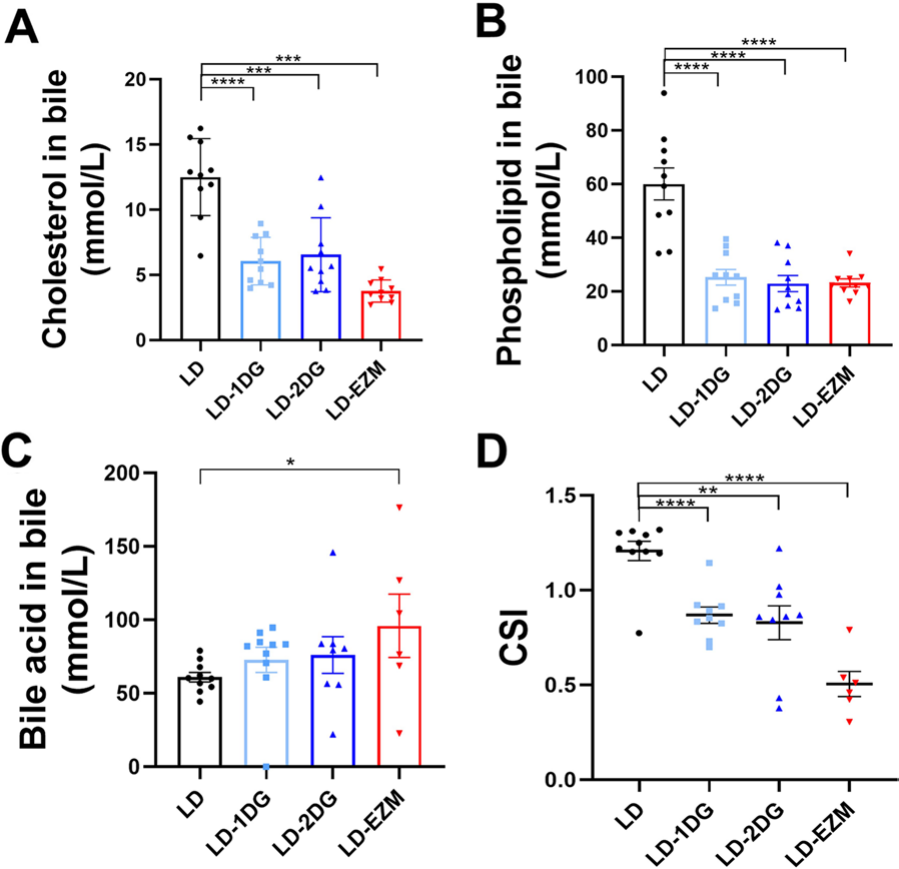
The influence of diosgenin on biliary lipid composition. **A**–**C** Biliary cholesterol, phospholipid and bile acid levels in each group, respectively. **D** Cholesterol saturation index (CSI) in each group. Data are expressed as mean ± SEM (n = 10 in each group in A-D). *P < 0.05, **P < 0.01, ***P < 0.001, ****P < 0.0001. LD: lithogenic diet; LD-1DG: LD + 1% diosgenin; LD-2DG: LD + 2% diosgenin; LD-EZM: LD + ezetimibe

In LD-1DG and LD-2DG groups, the cholesterol content in the mouse gallbladder bile was significantly lower (Fig. 2A). More prominent reduction was observed in LD-EZM group. Both DG and ezetimibe reduced biliary phospholipids levels to similar levels (Fig. 2B). The bile acid level seemed not to be affected in LD-DG groups, but increased in LD-EZM group (Fig. 2C). The cholesterol saturation index (CSI) was significantly reduced to be less than 1 in all three treatment groups (LD-1DG, LD-2DG and LD-EZM) (Fig. 2D).

To compare the secretion of biliary cholesterol from liver, common bile duct cannulation was performed to collect fresh hepatic bile. DG did not change hepatic bile secretion rate and ezetimibe slightly increased hepatic bile secretion rate (results not shown). Cholesterol concentration in fresh hepatic bile decreased dose-dependently in LD-DG and LD-EZM groups, though the difference was not statistically significant (Additional file 1: Fig S1).

### Diosgenin affects gene expression in cholesterol metabolism in liver and small intestine

**Fig. 3:**
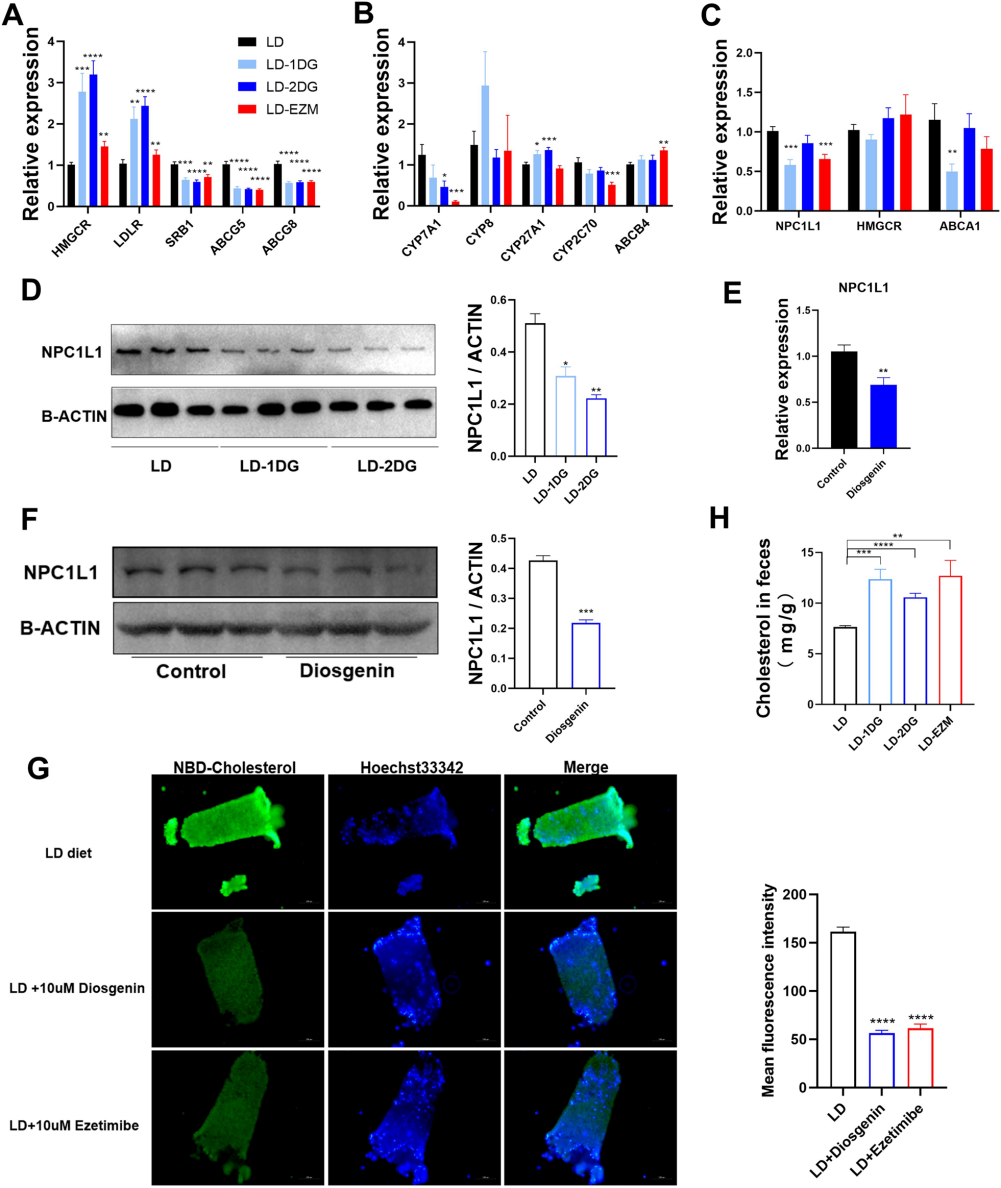
Diosgenin influenced the mice gene expression and the protein involved in cholesterol metabolism. **A** mRNA expression of genes involved in hepatic cholesterol metabolism. **B** mRNA expression of genes involved in hepatic bile acid and phospholipid metabolism. **C** mRNA expression of genes involved in intestinal cholesterol transportation. **D** Protein level of NPC1L1 in the intestine in mice from LD, LD-1DG, LD-2DG groups. The histogram shows gray value ratio of NPC1L1/β-ACTIN. **E** The mRNA expression of *Npc1l1* in small intestine from DG gavaged mice. **F** The protein level of NPC1L1 in the small intestine from DG gavaged mice. The gray value ratio was plotted on the side. **G** Fluorescence image showing cholesterol uptake in ex-vivo intestinal villi from mice fed with LD diet, LD + diosgenin diet or LD diet + ezetimibe. Mean fluorescence intensity statistics are plotted on the side. **H** Cholesterol content in feces from mice in each group. Data are expressed as mean ± SEM (n = 10 in each group in A, B; n = 5 in each group in E, F; n = 3 in each group in G). *P < 0.05, **P < 0.01, ***P < 0.001, ****P < 0.0001. LD: lithogenic diet; LD-1DG: LD + 1% diosgenin; LD-2DG: LD + 2% diosgenin; LD-EZM: LD + ezetimibe

Hepatic low-density lipoprotein receptor (*Ldlr*) and 3-hydroxy-3-methylglutaryl-CoA reductase (*Hmgcr*) was prominently up-regulated in LD-1DG, LD-2DG and LD-EZM groups compared with LD-only group (Fig. 3A). Scavenger receptor class B, type 1 (*Srb1*), however, decreased in all three groups. The cannalicular cholesterol transporters, ATP binding cassette subfamily G member 5/8 (*Abcg5/8*) decreased. No significant changes were observed for genes involved in bile acid biosynthesis in response to low dose DG (Fig. 3B). Cholesterol 7α-hydroxylase (*Cyp7a1*) was slightly inhibited by high dose DG and ezetimibe (Fig. 3B). No differences were observed in genes involved in phospholipid secretion.

Niemann Pick C1 like 1 protein (NPC1L1) is the key transporter for cholesterol absorption in the intestine. Its mRNA expression decreased in low DG group, though not in high DG group (Fig. 3C). At the protein level, DG dose dependently reduced the NPC1L1 proteins in proximal intestines from mice in both DG groups (Fig. 3D). To further verify the inhibitory effect of on mouse *Npc1l1* expression by high DG, we measured the mRNA and protein levels in mice gavaged with high DG. The results showed that DG reduced the transcription of *Npc1l1* in small intestine (Fig. 3E) as well as its protein level (Fig. 3F). To reduce the detection error, another 3 pairs of primers targeting *Npc1l1* gene were designed. The reproducible results could be obtained (Additional file 1: Fig. S2).

In mice fed with DG and ezetimibe group, less absorption of NBD-cholesterol in their proximal intestine mucosa was shown (Fig. 3G). This indicating an inhibition of intestinal cholesterol absorption in mice fed with DG. In supporting, feces from mice in LD-1DG/LD-2DG and LD-EZM groups all showed increase cholesterol content (Fig. 3H).

### Diosgenin inhibited cholesterol absorption in Caco2 cells by reduced NPC1L1

**Fig. 4:**
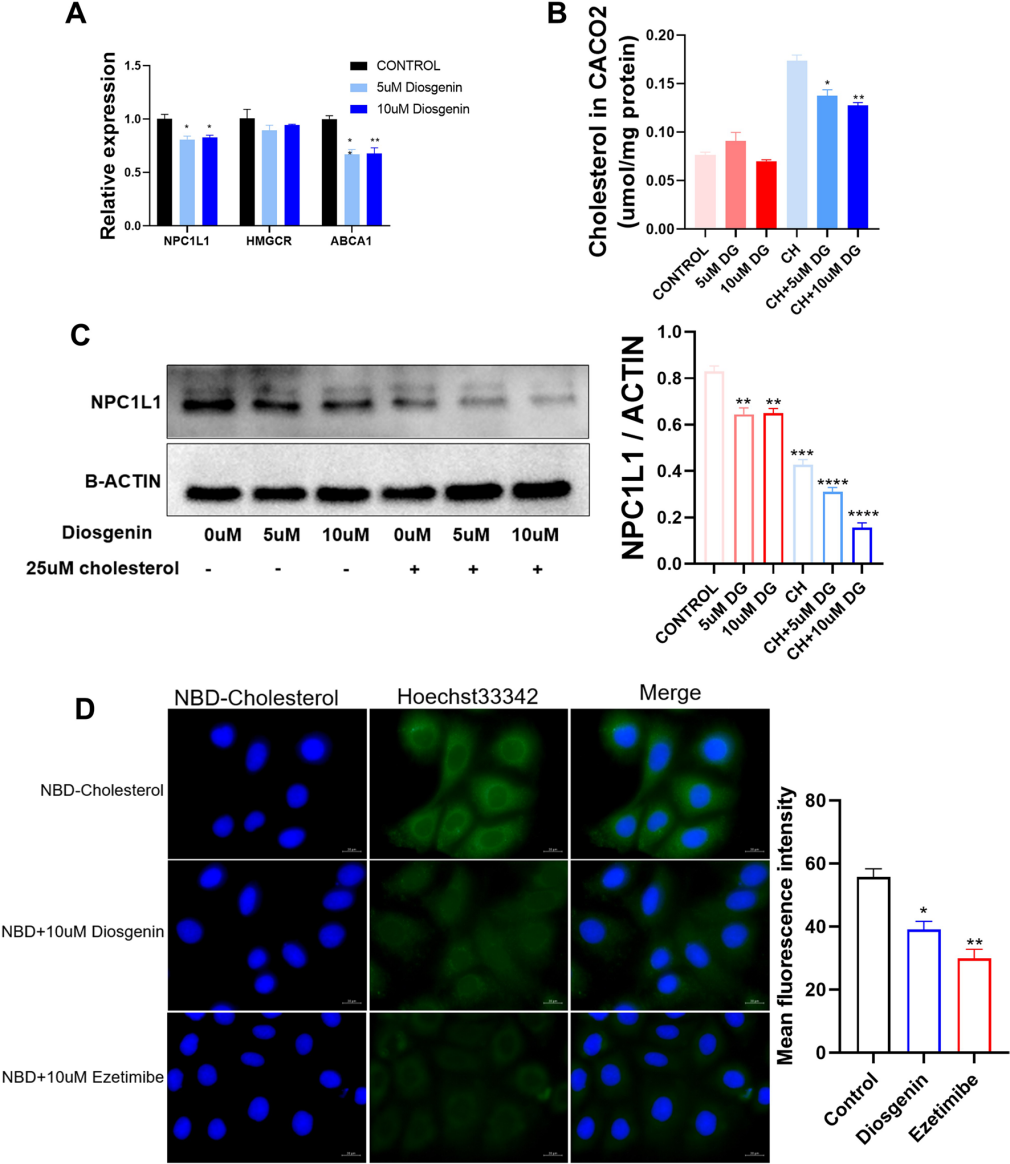
Diosgenin inhibited cholesterol absorption in Caco2 cells. **A** mRNA expression of genes in Caco2 cells under diosgenin treatment. **B** Cholesterol level in Caco2 treated with diosgenin or cholesterol. **C** Protein level of NPC1L1 in Caco2 cell treated with diosgenin or cholesterol. The gray value ratio was plotted on the side. **D** Fluorescence image of cholesterol uptake in Caco2 cells under NBD-cholesterol containing medium with and without diosgenin and ezetimibe. Mean fluorescence intensity statistics are plotted on the side. Data are expressed as mean ± SEM (3 repeats per experiment). *P < 0.05, **P < 0.01, ***P < 0.001, ****P < 0.0001

To further investigated the mechanism of inhibition on cholesterol by DG, we treated the Caco2 cells with/without cholesterol. DG was shown to decrease *Npc1l1* mRNA expression in Caco2 cells (Fig. 4A). Consistently, the protein levels of NPC1L1 and cholesterol contents in cells were lower under DG treatment especially under cholesterol treatment (Fig. 4B, C). Using NBD-labeled cholesterol, DG and ezetimibe was shown to inhibit cholesterol uptake by Caco2 cells (Fig. 4D). Collectively, these data indicated that DG inhibited cholesterol absorption in intestinal cells by reducing expression of NPC1L1.

### Diosgenin decreases the expression of NPC1L1 by repressing the transcriptional activity of STAT3

**Fig. 5:**
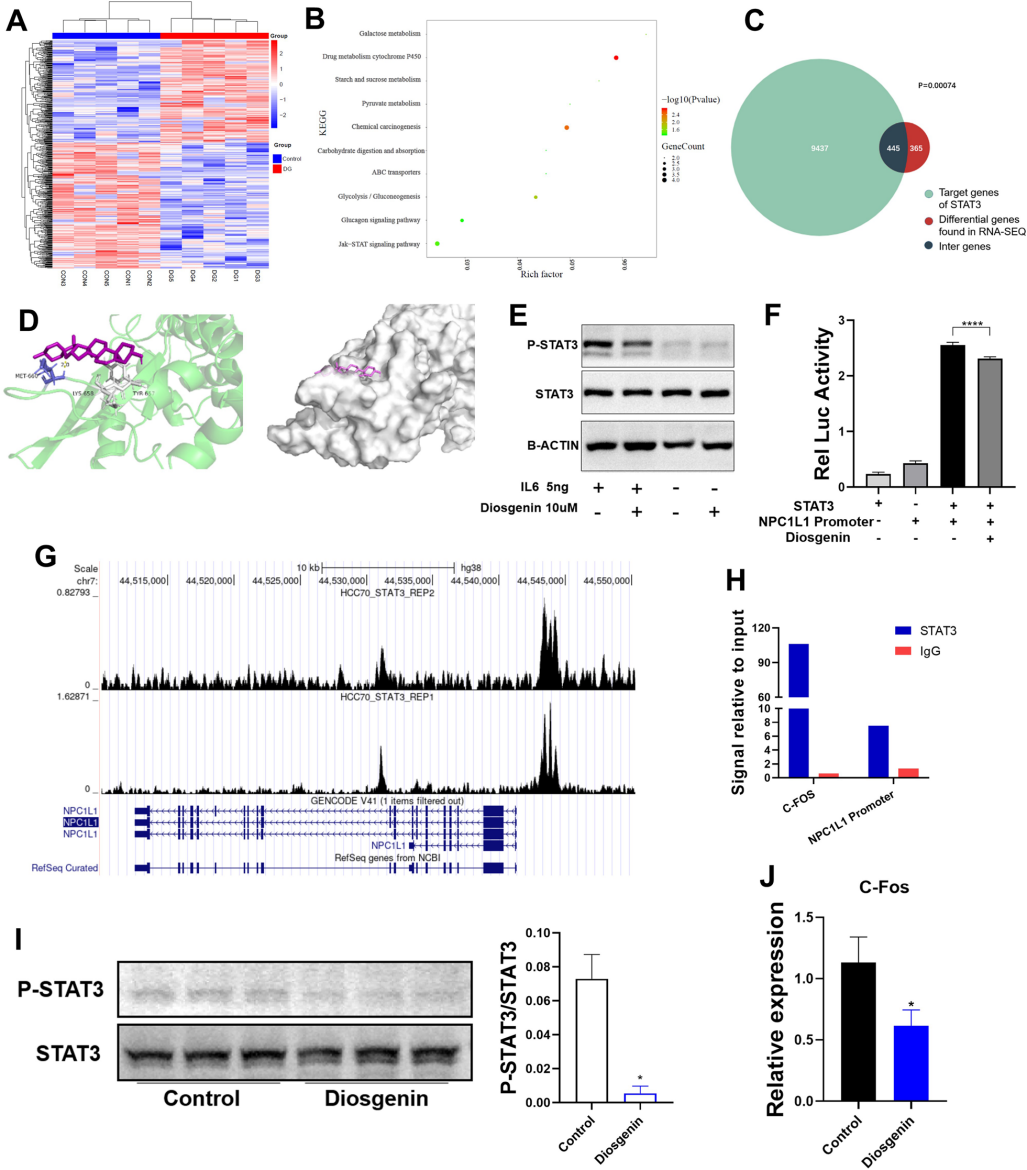
STAT3 involved in the downregulation of NPC1L1 by diosgenin. **A** Heatmap show of differentially expressed genes found in RNA-Seq between diosgenin treated Caco2 cells and the control cells. **B** Bubble chart of differentially expressed genes KEGG enrichment. **C** Venn diagram demonstrated the intersections of genes between differentially expressed genes and STAT3 target genes. **D** Predicted 3D structures of STAT3 protein in interaction with diosgenin. **E** The effect of IL6 and diosgenin on STAT3 phosphorylation. **F** The NPC1L1 promoter activity was measured by firefly luciferase activity and normalized by renilla luciferase activity. STAT3 overexpression plasmid was induced to enhance the NPC1L1 promoter activity. **G** Analysis of Chromatin immunoprecipitations (CHIP)-Seq data of STAT3 from published database. The peaks ahead of NPC1L1 RefSeq referrred to enrichment of STAT3 target sequence. **H** CHIP assay was performed with IL6 treated in Caco2 cells. The enriched DNA was quantified by real-time PCR. Human c-Fos promoter was used as positive control. The result was represented as signal relative to the total amount of input chromatin and adjusted by IgG group which was equivalent to one. **I** Intestinal STAT3 phosphorylation of DG gavage mice compared to control group by Western blot. The gray value ratio was plotted on the side. **J** The mRNA expression of STAT3 downstream gene c-Fos in small intestine from DG gavage mice compared to that from the control group. Data are expressed as mean ± SEM (n = 5 in each group). *P < 0.05, ****P < 0.0001

To further explore how DG reduces NPC1L1 expression, RNA-seq was performed in Caco2 cells treated with and without DG. Gene expression difference showed distinct differences between the two groups (Fig. 5A). KEGG enrichment indicated enrichment of JAK/STAT signaling pathway under DG treatment (Fig. 5B). Among the 810 genes with FDR < 0.01 obtained by further screening, over half (445/810) were target genes of STAT3 (Fig. 5C). Molecular docking suggested the interaction between DG and STAT3. The maximum predicted binding energy of DG with STAT3 was − 5.69 kcal/mol and one hydrogen bond formed at residue MET-660 in the active pocket (Fig. 5D).

IL6 is an activator of STAT3 phosphorylation [22]. DG treatment was shown to decrease the phosphorylation of STAT3 proteins in Caco2 cells under basal condition and under IL6 induction (Fig. 5E).

Then, we constructed dual-luciferase reporter gene containing NPC1L1 promoter. STAT3 could apparently induce the transcriptional activity of NPC1L1 promoter, which could then be inhibited after adding DG (Fig. 5F). Using publicly available CHIP-Seq database analysis [23], enrichment peaks of STAT3 at the promoter region of NPC1L1 were identified (Fig. 5G). Furthermore, using CHIP assay, we found enrichment of STAT3 binding at NPC1L1 promoter regions (Fig. 5H). We also confirmed this phenomenon in vivo. Intestine samples from DG gavaged mice were used to check its effect on STAT3 signal. DG decreased the phosphorylation of STAT3 as well as the expression of c-Fos, a known downstream protein of this signaling pathway (Fig. 5I, J).

### Diosgenin modified gut microbes

**Fig. 6:**
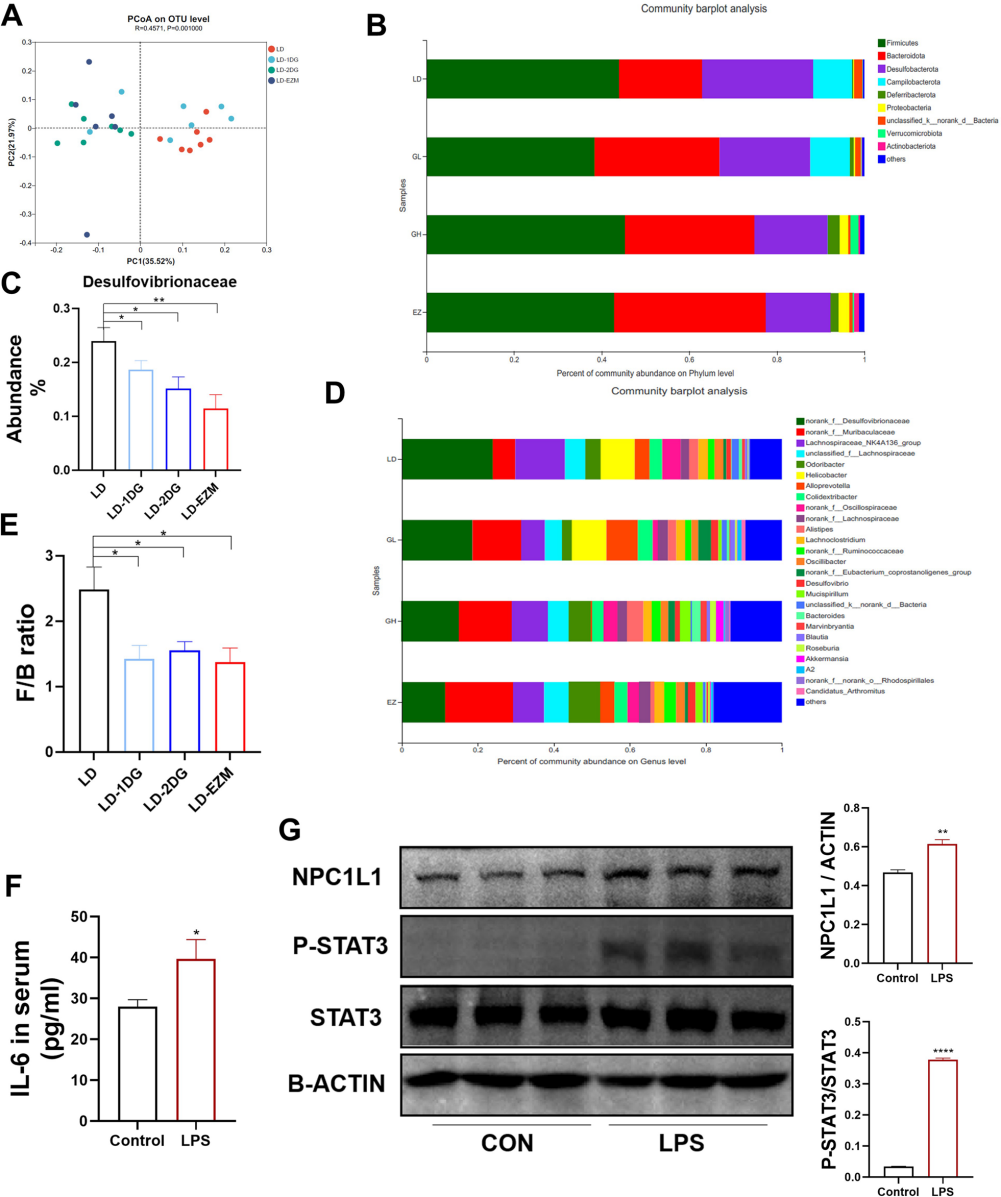
Diosgenin altered gut microbes in mice. **A** Gut microbiota data of four groups were visualized by principal co-ordinates analysis. **B** The bacterial composition of the four groups at the phylum level. **C** The abundance of *Desulfovibrionaceae* in the four groups. **D** The bacterial composition of the four groups at the genus level. **E** *Firmicutes/Bacteroidetes* ratio of the four groups. **F** Serum IL6 in mice injected with LPS. **G** Intestinal expression of protein levels of NPC1L1 and STAT3 phosphorylation in mice injected with LPS. The gray value ratio was plotted on the side. Data are expressed as mean ± SEM (n = 7 in each group in A-E, n = 5 in each group in **F**, **G**). *P < 0.05, **P < 0.01, ****P < 0.0001. LD: lithogenic diet; LD-1DG: LD + 1% diosgenin; LD-2DG: LD + 2% diosgenin; LD-EZM: LD + ezetimibe

At last, we performed 16S RNA sequencing on ceceal microbiota composition to monitor the any effect of DG on modification of gut microbiota. DG showed a separated pattern in microbiota in mice from LD-only on principal co-ordinates analysis (PCoA). (Fig. 6A) The three bacteria with the highest abundance at the phylum level were *Firmicutes*, *Bacteroidota* and *Desulfobacterota* (Fig. 6B). At genus level, we found *Desulfovibrionaceae* which belongs to *Desulfobacterota* phylum and was the main genus in LD group, gradually decreased in LD-1DG, LD-2DG, LD-EZM groups (Fig. 6C, D). Similarly, the *Firmicutes/Bacteroidetes* (F/B) ratios, a biomarker of gut–microbe balance, were reduced in LD-1DG, LD-2DG, LD-EZM (Fig. 6E). To demonstrate the significant influence of bacterial endotoxin on STAT3 signaling, mice were intraperitoneal injected with LPS. As expected, serum IL6 increased in LPS group (Fig. 6F), which subsequently led to the increase of STAT3 phosphorylation in the small intestine, and the increase of NPC1L1 protein accordingly (Fig. 6G).

## Discussion

**Fig. 7:**
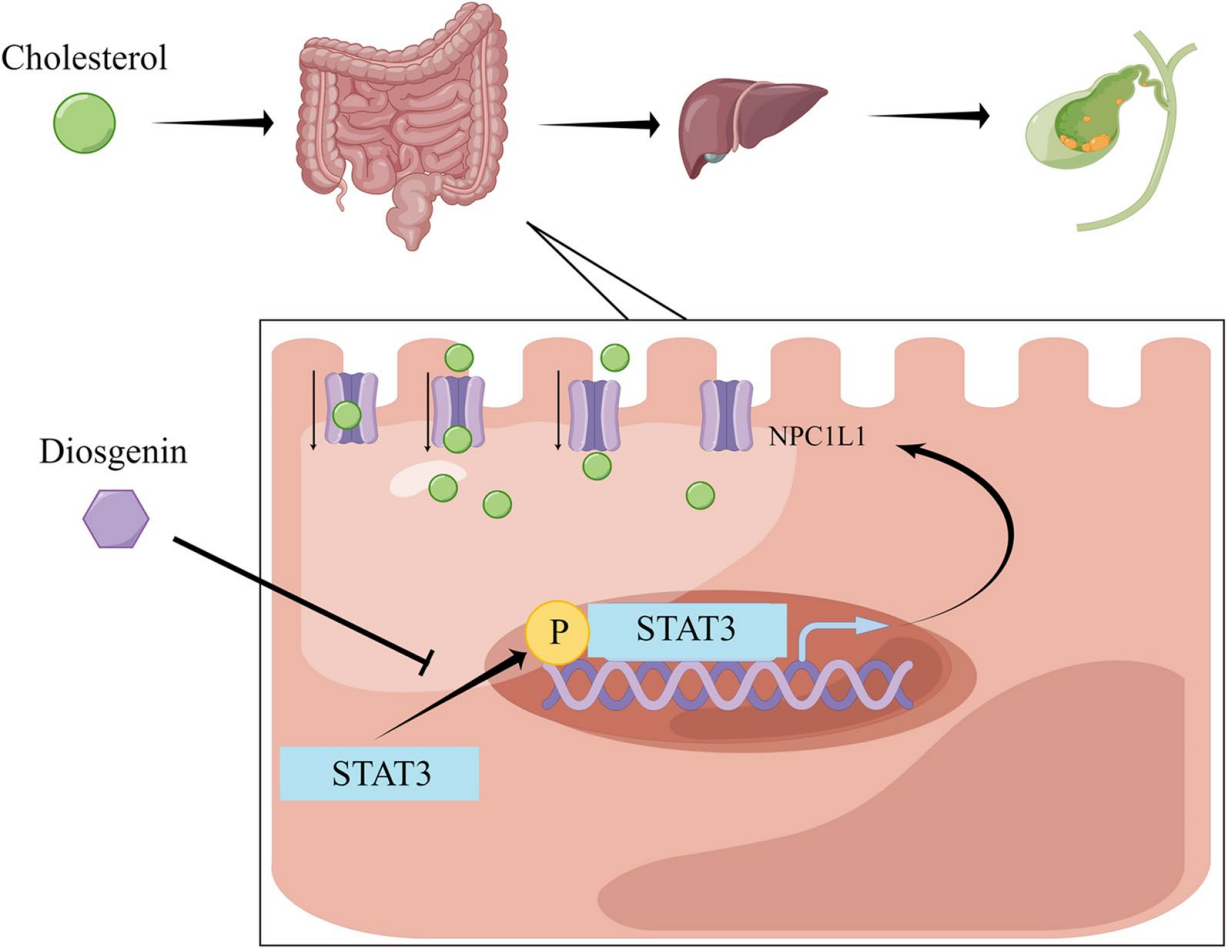
The schematic diagram showing the mechanism on DG prevent cholesterol gallstone formation by regulating intestinal NPC1L1 expression

In the present study, we found that DG decreased incidence of cholesterol gallstone in mice by lowering NPC1L1 expression to inhibit intestinal cholesterol absorption (Fig. 7). This effect was achieved through inhibiting phosphorylation of STAT3 mediated transcriptional regulation of NPC1L1 expression in the intestinal absorptive cells. Our results, for the first time, provided evidences for DG to be a promising drug for preventing cholesterol gallstone formation.

Clinical and experimental studies have found significant relationship between intestinal cholesterol absorption and cholesterol gallstone formation [6, 24]. Targeted inhibition of intestinal cholesterol absorption, e.g. using ezetimibe, has been reported to reduce intestinal cholesterol uptake and prevent gallstone formation [7], which was also confirmed in the present study. In our study, we found dietary DG intake could reduce serum cholesterol levels due to its potential role in inhibiting intestinal cholesterol absorption. Thus, DG was shown to provide a novel way to prevent gallstone formation. Another way to regulate intestinal cholesterol absorption is to modify bile acid composition and hydrophobic index of bile acids, which are important determinant for cholesterol containing micelle formation and intestinal absorption [25]. Higher bile acid hydrophobicity promotes cholesterol transport to villi [25, 26]. Knockout of cholesterol 12α-hydroxylase (*Cyp8b1*) gene, one of the key enzymes in bile acid synthesis, could alter bile acid profile by lowering cholic acid production can inhibit intestinal cholesterol absorption and prevent hypercholesterolemia and gallstone formation [27]. In the present study, we found no effects on bile acids by DG intake.

NPC1L1 is presently considered as a key protein for intestinal absorption of cholesterol. We found DG inhibiting intestinal cholesterol absorption through its transcriptionally regulation of NPC1L1 expression. So far, the reported transcriptional regulator of NPC1L1 included PPARα and LXR [28]. DG and its derivatives are reported to inhibit the activation of STAT3 into its phosphorylated form [29, 30]. Our work elaborated the role of DG on NPC1L1 transcriptional regulation by STAT3. Luciferase reporter assay confirmed the ability of STAT3 to activating NPC1L1 gene transcription. Using CHIP assay, the binding of STAT3 at the promoter region of NPC1L1 gene was further confirmed in consistent with data using CHIP-Seq database analysis [23]. The above conclusions have also been verified in animal model. In contrast, these regulations could be inhibited by adding DG. STAT3 dimerized by phosphorylation at Tyr705, bound with DNA through SH2 domain, resulting in transcriptional activity [31]. According to the molecular docking, DG as ligand bound to Met660 and obscured Tyr657 that is important for transcriptional activity [32]. To our knowledge, the present study for the first time provided evidences for STAT3 as transcription factor for NPC1L1. Chen et al. found activation of STAT3 promotes the expression of the cholesterol transporter ABCA1 in macrophage [33]. Activation of ABCA1 also counteracts STAT3 [34], which formed a positive feedback loop. Since STAT3 was not constitutively activated under physiological conditions, we only observed the inhibitory phosphorylation of DG under the stimulation of IL6. Lithogenic diet induced inflammatory responses a could lead to elevation of IL6 and an activation of STAT3. Both cholesterol and cholic acid were proven to activate the JAK/STAT3 pathway [35, 36].

Gut microbiota may be another target of DG. We showed significantly improvement of the alteration of gut microbiota under lithogenic diet by DG. The decrease of F/B ratio and the *Desulfovibrionaceae* may be the two major features of the effect by DG. Gallstone, as a metabolic disease, treated with therapeutic drugs might reduce gut microbiota F/B ratio [10, 37]. *Desulfovibrionaceae*, is anaerobic, gram-negative bacterium, which utilizes certain fatty acids or as carbon sources to reduce sulfate to H_2_S. It was able to modulate bile acid profiles to be more hydrophobic and promoting biliary cholesterol secretion through H_2_S signal [26]. On the other hand, *Desulfovibrionaceae* could produce endotoxins and pro-inflammatory cytokines such as IL6 [38], a potent agonist of the JAK/STAT3 pathway. Dysbiosis gives the intestinal mucosa greater exposure to LPS, and subsequent IL6 and STAT3 activation may lead to increased cholesterol absorption. In the context of increased STAT3 phosphorylation, DG therapy may be more effective, it suppressed the scale of *Desulfovibrionaceae* and also may indirectly down-regulate the STAT3/NPC1L1 pathway.

The processing of hepatic cholesterol into bile is a key step in controlling the formation of gallstones. Down-regulation of ABCG5/8 in response to hepatic cholesterol pool alleviated cholesterol loading into bile and decreased cholesterol saturation index of gallbladder bile. Though CYP7A1, was reduced by DG in liver; while CYP27A1 was up-regulated, the amount of bile acid did not differ between group of mice. Lastly, as a common ingredient in the daily diet, long-term oral DG was shown to be safe and tolerable. No mice in our study displayed any symptoms of organ damage.

## Conclusion

Our present study proved that dietary supplement with DG could prevent cholesterol gallstone formation in mice. DG could inhibit phosphorylation of STAT3, in turn, decreasing the expression of NPC1L1 at the transcription level. Whether DG could be a promising drug to prevent gallstone formation in human needs further study.

## Supplementary Information

**Additional file 1: Fig. S1.** Cholesterol content in fresh bile of LD, LD-1DG, LD-2DG and LD-EZM groups.LD: lithogenic diet; LD-1DG: LD + 1% diosgenin; LD-2DG: LD + 2% diosgenin; LD-EZM: LD + ezetimibe. **Fig. S2.** Intestinal *Npc1l1* mRNA expression of DG gavaged mice measured with another 3 different pairs of primers.. * P < 0.05.

## Data Availability

The datasets generated during the current study are available in SRA repository, ID: PRJNA922754 and PRJNA923121. (https://www.ncbi.nlm.nih.gov/bioproject/PRJNA923121/); (https://www.ncbi.nlm.nih.gov/bioproject/PRJNA922754/).
