# Supplementary figures and images for: Dietary diosgenin transcriptionally down-regulated intestinal NPC1L1 expression to prevent cholesterol gallstone formation in mice
Source: J Biomed Sci. 2023 Jun 27;30:44. doi: 10.1186/s12929-023-00933-3 (PMC10294472; doi:10.1186/s12929-023-00933-3)

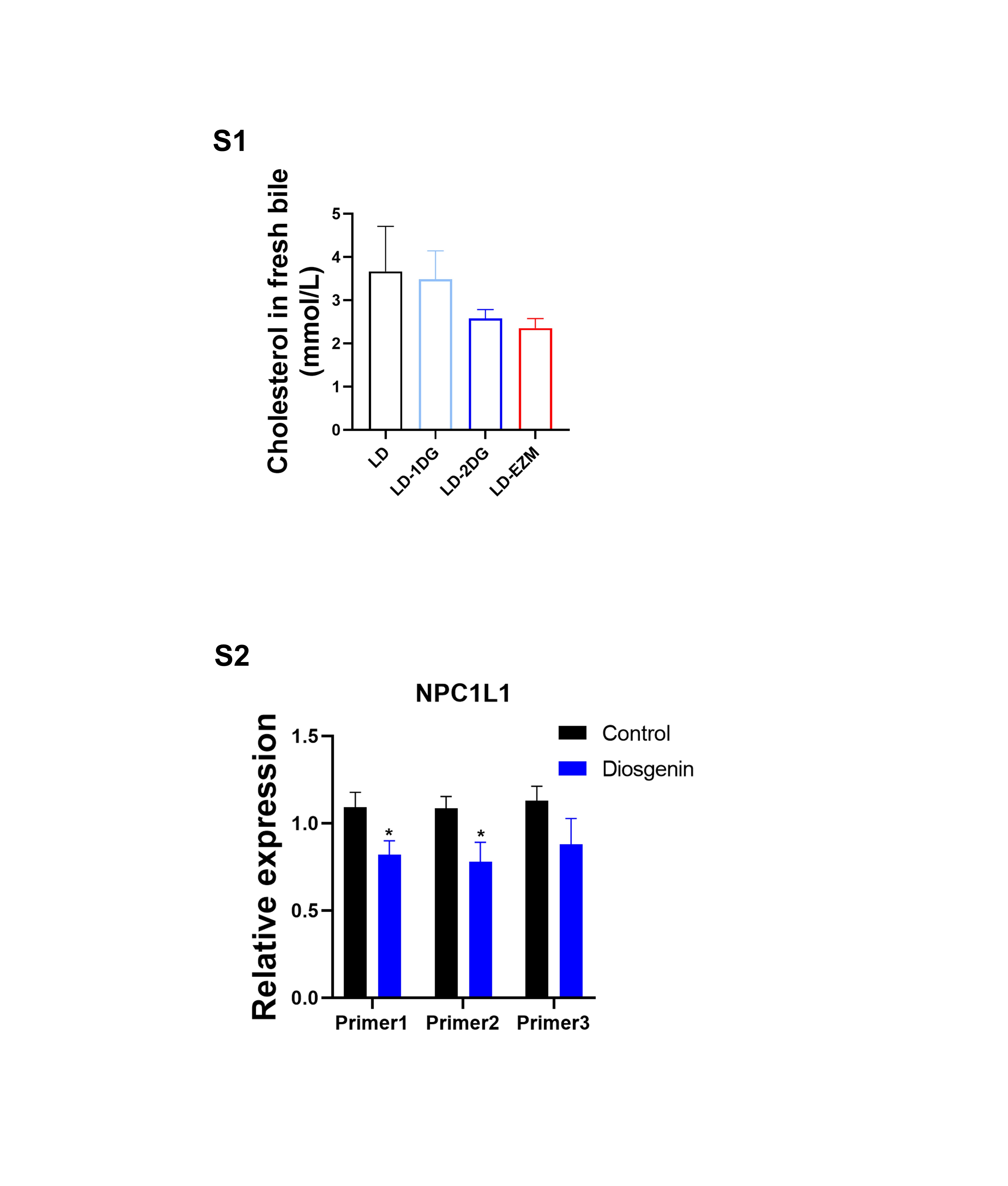

Supplement: Supplementary file 1 — Additional file 1: Fig. S1. Cholesterol content in fresh bile of LD, LD-1DG, LD-2DG and LD-EZM groups.LD: lithogenic diet; LD-1DG: LD + 1% diosgenin; LD-2DG: LD + 2% diosgenin; LD-EZM: LD + ezetimibe. Fig. S2. Intestinal Npc1l1 mRNA expression of DG gavaged mice measured with another 3 different pairs of primers.. * P < 0.05. [file 12929_2023_933_MOESM1_ESM.jpg]
